# Exploring the Acceptability of the STOP Method for Addressing Weight Loss Misinformation on Social Media: An Interview Study

**DOI:** 10.1002/osp4.70080

**Published:** 2025-06-21

**Authors:** Danielle E. Jake‐Schoffman, Chrishann Walcott, Hannah A. Lavoie, Francesca Wilkins, Megan A. McVay, Montserrat Carrera Seoane

**Affiliations:** ^1^ Department of Health Education & Behavior University of Florida Gainesville Florida USA; ^2^ Center for Integrative Cardiovascular and Metabolic Disease University of Florida Gainesville Florida USA

**Keywords:** misinformation, online information seeking, social media, weight loss

## Abstract

**Background:**

Adults attempting weight loss often seek information online, though high prevalence of health misinformation. We aimed to gather feedback on a novel video‐based approach to support adults in navigating weight‐related misinformation online.

**Methods:**

We developed three brief videos presenting our novel mnemonic approach, the **STOP** method: Is someone trying to **S**ell you something? Does it sound **T**oo good to be true? Is it **O**ut of step with messages from trusted sources? Does it focus on fast **P**rogress? Adults with BMI ≥ 25 kg/m^2^ interested in weight management strategies provided feedback on the videos via semi‐structured interviews. Interviews were transcribed verbatim and analyzed using an emerging theme approach.

**Results:**

Participants (*N* = 14) were 64.3% female, 57.1% non‐Hispanic white, with a mean age of 44.6 ± 18.0 years and BMI 31.5 ± 4.3 kg/m^2^. Interviews revealed several themes. Participants found the STOP method to be accessible and would recommend it to others. They felt the videos effectively introduced the STOP method and its application and had minor suggestions for improved clarity and suggestions regarding visual appeal and ways to promote video engagement.

**Conclusions:**

The STOP method was acceptable and the brief videos were well‐received; the results will direct video refinement and further testing.

## 1 Introduction

Almost half (49%) of US adults have attempted to lose weight within the last year [1]. While many individuals seek professional guidance for weight management, a growing number instead choose self‐directed approaches, often using the internet for health information (e.g., weight loss strategies) [2]. In fact, 46.1% of adults report that they have looked for health information online within the past 12 months [3].

As digital communication continues to evolve, how individuals seek health information has significantly transformed, resulting in the availability of vast amounts of information on different media platforms, including potentially harmful information. The rise of social media has amplified the trend in online health information seeking, with platforms like Facebook, Instagram, and TikTok becoming dominant sources of health information, including weight loss guidance [4, 5]. While social media and online communities have successfully been used in behavioral weight loss interventions [2, 5, 6, 7, 8], the high volume of health information online often exposes users to false, inaccurate, or incomplete health information and advice, referred to as misinformation [9]. Misinformation regarding weight management is particularly prevalent, ranging from fad diets and misleading product claims to potentially harmful “quick fix” solutions [10, 11]. Recent research has raised significant concerns about the prevalence of weight management misinformation online, identifying it as a potential barrier to successful weight loss [10, 11, 12, 13], and raising concerns about how well people will be able to discern credible advice to aid their weight loss efforts on their own [10, 14].

Previous research has explored the prevalence of health misinformation online [15, 16, 17, 18], and more recently, a variety of individual‐focused approaches to addressing online misinformation have been developed and tested, from pre‐emptive inoculation to teaching verification strategies [19]. However, to our knowledge, few studies have focused on applying these to weight loss and nutrition related misinformation and the lack of standardized tools or training to aid individuals in this process may leave them to rely on their judgment or poorly enforced community guidelines [14]. Notably, factors such as lower levels of education, reduced health literacy, greater distrust in the healthcare system, and a preference for alternative medicine leave certain individuals more suspectable to believing misinformation [14]. Of further concern, individuals who are susceptible to online information about one health topic may be more vulnerable to misinformation across various health topics [14]. This highlights the urgent need for user‐friendly, accessible, and reliable educational resources to empower individuals to critically evaluate online health information, both independently and within behavioral interventions, enabling them to make informed decisions about their weight management journey.

The primary aim of this study was to develop and pilot test brief educational videos aimed at supporting adults in identifying and navigating weight management misinformation online. Overall, the objective of the present study was to educate participants on misinformation, understand participants' thoughts and feedback on the videos using our novel mnemonic approach (i.e., the STOP method), and explore if the videos impacted participants' (short term) confidence in identifying and handling misinformation.

## 2 Methods

### 2.1 STOP Method and Video Development

As a preliminary step, three brief videos were developed using evidence‐based strategies to educate adults about weight‐related health misinformation and how it can appear on social media. Educational videos were chosen considering their accessibility to a larger audience and ease of dissemination, especially in the primary care context. The videos outline the **STOP** Method, a brief heuristic tool designed to provide people with a quick and easy tool to use if they suspect they have found misinformation. The tool was based on the format of a general misinformation tool, the “CRAAP Test” [20] that was developed by librarians at California State University, Chico in 2004 and has since been used by many university librarians to help first‐year college students learn about finding good sources of information online. The CRAAP method uses a catchy and easy‐to‐remember name to help people remember five evaluation criteria by which to assess potential misinformation [21]. While not empirically tested, the CRAAP method has been widely used with good anecdotal results [21]. Thus, the STOP method was developed using this model but with a specific focus on the types of health misinformation most commonly observed in the context of weight loss groups online.

The **STOP** method stands for:

- Is someone trying to **S**ell you something?
- Does it sound **T**oo good to be true?
- Is it **O**ut of step with messages from trusted sources?
- Does it focus on fast **P**rogress?

**FIGURE 1:**
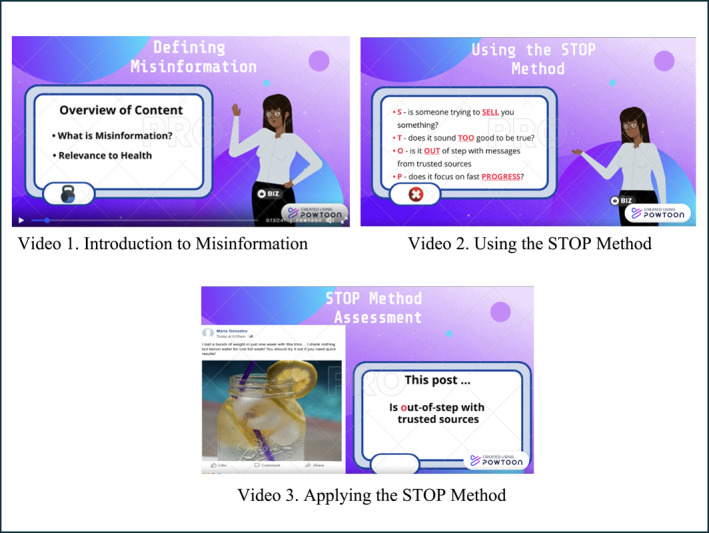
Screenshots from the STOP method videos.

Video 1, “Introduction to Misinformation” (2.5 min long), introduces and defines “misinformation” to viewers, as well as why misinformation is relevant amidst the common use of social media to find reliable information on a daily basis. See Figure 1 for screenshots from the three videos. Video 2, “Using the STOP Method” (4.5 min long), outlines the “STOP Method” as a tool for assessing the information encountered on social media. Each of the four components of “STOP” is explained thoroughly after introduction. Video 3, “Applying the STOP Method” (5 min long), presents a brief recap of the STOP method and instructs viewers on applying the STOP Method to social media by presenting mockup Facebook posts that contain examples of unreliable content that individuals might encounter. The narrator instructs viewers to read the posts and then choose possible letters from the STOP Method that might apply to the posts.

### 2.2 Participants and Recruitment

Participants were adult community members, aged 18–75 years, with a Body Mass Index (BMI) ≥ 25 kg/m^2^, interested in learning weight management strategies, could read and understand English without a translator, and had access to a stable internet connection on a mobile device or computer [22]. Participants were recruited through ResearchMatch and organic social media posts on Twitter and Facebook. Recruitment materials directed potential participants to a REDCap eligibility questionnaire. Eligible individuals were contacted by researchers to invite them to complete the interview via Zoom. The target sample size was *N* = 10–15 adults, estimated based on previous research demonstrating that themes identified during in‐depth interviews can reach saturation around *N* = 12 respondents [23].

### 2.3 Data Collection

Participants took part in a one‐hour recorded interview conducted via Zoom audio. The interview began with the researcher asking two questions, developed for this study, where the participant was asked to rate their confidence on a scale from 1 to 7, with 1 being “not at all confident” and 7 being “very confident” to: “I am confident that I can recognize or identify if health information I see online is false or inaccurate” and “I am confident that I know what to do if I see health information online that is false or inaccurate.”

A semi‐structured interview script was used to first explore participants’ experiences with health misinformation online and their comfort with identifying and handling it (see Supporting Information S1 for full interview guide). Next, participants were asked to watch each of the STOP Method videos, first without interruption and then with specific probes to gather feedback on the acceptability of the STOP Method in general as well as the way it was specifically presented in the videos. The interview also aimed to gather feedback to inform future improvements to the video presentation and content, with the goal of ensuring that the videos are appealing and understandable to a larger, diverse audience. Further, as a future goal is to embed the videos in a primary care‐based weight loss intervention, the interview explored the impact of delivering the materials in that context.

After the interview, participants were asked demographic questions and basic questions about their social media use and use of online weight loss communities. Participants were compensated $20 for the study visit.

### 2.4 Data Analysis

Interviews were transcribed verbatim. Analysis followed the principles of the constant comparative method [24, 25]. HAL, CW, and FLW first familiarized themselves with the interviews and reviewed early analytical memos/notes to develop initial themes for a codebook. They then independently coded three interviews, where emerging themes were added, and the codebook was refined. After coding six interviews, HAL, CW, and FLW met to iteratively develop codes and refine the codebook. A single coder (CW) then used the refined codebook to code the remaining transcripts and revisited initially analyzed transcripts to ensure consistency of coding/interpretation. All data were then organized and merged into a coding matrix based on the emerging themes.

The study was approved by the University of Florida Institutional Review Board (#ET00020974).

## 3 Results

### 3.1 Descriptive Characteristics of Sample

Semi‐structured interviews were conducted with *N* = 14 participants. Participants were on average 44.6 ± 18.0 years old (range 20–70 years) with a BMI of 31.5 ± 4.3 kg/m^2^. Over two‐thirds of the sample (64.3%) identified as female; more than half of the sample identified as white (57.1%), 28.6% as Black or African American, and 14.2% identified as Asian or American Indian or Native American. Educational status varied; nearly three‐quarters of participants had obtained at least a bachelor's degree (71.4%), while others had an associate degree (14.3%) or some college‐level education, including technical school (14.3%). Additional demographics are included in Table 1.

**TABLE 1:** Sociodemographic characteristics, social media use, and misinformation confidence (*n* = 14).

	*n* (%), mean (SD)
Gender	
Female	9 (64.3)
Male	5 (35.7)
Age, in years	44.6 (18.0)
BMI, kg/m^2^	31.5 (4.3)
Education level	
Some college	2 (14.3)
Associate's degree	2 (14.3)
Bachelor's	10 (71.4)
Race/Ethnicity	
White	8 (57.1)
Black or African American	4 (28.6)
American Indian or Alaska native or Asian	2 (14.2)
Marital status	
Single	4 (28.6)
Divorced	4 (28.6)
Married	4 (28.6)
Living with partner or other	2 (14.3)
Employment status	
Full‐time	6 (42.9)
Part‐time	4 (28.6)
Retired or student	4 (28.6)
Frequency of using smartphone to find health‐related information	
A couple times a week	6 (42.9)
More than once a day	2 (14.3)
Rarely	1 (7.1)
Uses social media to find weight loss information	
Yes	7 (50.0)
About once a day	1 (7.1)
Once a week (1–2 days)	1 (7.1)
A few times a week (3–5 days)	3 (21.4)
Every few weeks	2 (14.3)
No	7 (50.0)
Social media platforms used to find weight loss information	
Facebook	3 (21.4)
YouTube	2 (14.3)
Instagram	2 (14.3)
Twitter/X	0 (0.0)
Snapchat	0 (0.0)
Other or None	2 (14.3)
Reads or comments on social media sites about weight loss	
Yes	8 (57.1)
No	6 (42.9)
Confidence in recognizing or identifying health misinformation	
Before videos	5.3 (1.4)
After videos	6.1 (0.8)
Confidence in handling health misinformation	
Before videos	5.2 (1.9)
After videos	5.8 (2.0)

Most participants (92.9%) reported using social media to find health‐related information, including Facebook (71.4%), YouTube (57.1%), and TikTok (21.4%). Half of the participants used social media to find information on weight loss at least once a month, and half also reported having read or interacted with social media websites where users had the ability to share anecdotes related to weight loss and similar topics.

### 3.2 Interview Themes

Themes identified from the interviews encompassed participants' confidence in identifying and handling misinformation, thoughts about video presentation and spoken delivery, and feedback on overall appeal, content, message, and video presentation aesthetics. Overall, participants expressed positive feedback on the STOP Method videos and offered valuable insights for researchers to consider in refining future video deliveries. Interview themes are summarized below and in Table 2.

**TABLE 2:** Qualitative themes, sub‐themes, and exemplary quotes.

Theme	Sub‐theme	Participant quotes
Self‐efficacy and confidence in identifying misinformation	Increase in self‐efficacy	“Well I do. Yeah I do. I think that um, I yeah, definitely … You know, a little bit more of an understanding of what to look for. And uh, yeah.”—ID15
	Pre‐existing confidence	“So I'd say I'm pretty confident in what I feel is the best best choice for health. However, sometimes I'm a little skeptical of what information is being told […] I see a lot of physicians now that are on social media. And I'm a little skeptical of that. This is gonna sound horrible that they have all this time to spend on social media, ‘cause it just seems conflicting, ‘cause. That's not what I see if I'm in the actual practice in my doctor's office. I'm like, there's my personal doctors don't have time for that.”—ID8
	Confusion on “handling” or “Dealing with” misinformation	“Yeah, I mean, I probably again, I'd probably say the same as before. I Think I said seven before, and I'd still say seven. I Just kinda like know to not engage with it. Really like I guess if, like, there's some specific action you want folks to take with regard to like reporting, misinformation or whatnot, then then maybe that isn't quite clear, but like to me, it just like, oh, like can't really … there's too much out there to just like get rid of it all. Just…people have to learn how to sift through it.”—ID4
	Handling misinformation	“Yeah, I think it. It improved it. It gives it, it reminds somebody to like it says, ‘STOP’ and before you get kind of pulled in.”—ID8
Reception to and perception of the STOP method	Overall approval	“No, I think it was was really well for your target audience, and I like the STOP procedure, was kind of a good ideal, something easy to remember with you know things that point out each of each of the different factors that will alert you to inaccurate information. So I thought that was a, it was a good strategy.”—ID250
	Ease of use	“Just the stop method and the suggestion. I Really I can't think of any other method that I used um or that I'm going to use better than that, so that was a pretty smart move.”—ID20
	Willingness to recommend to others	“Yeah, it's digestible. I think you could easily put on a rack card as well, the information that you all did have on the other slides as far as the STOP method. I Think that'll be good information to have like on a slide deck card that could be posted in health clinics across the country, or even the state …”—ID235
	Concerns about applicability to others	“If they were more geared toward losing weight to better your health or losing weight for, women who've gone through menopause, or for senior citizens something that was more geared for my age, and where I am in my life as opposed to for everybody.”—ID9
	Suggestions for improvement	“I don't know. Just like seeing out of message … like the wording. It kind of caught me off guard, I think like, were you trying to say that if it doesn't align previous medical? You know, studies? Is that what you're trying to say? Yeah. I don't know … And so to me when I read that, like it took me a little bit until you like mentioned some examples, but I don't know. I could be understood by others.”—ID12
Video content and message	Desire for evidence‐based strategies	“… I think I'll definitely use it. I mean, I've I'm kind of using been using something of a strategy like that, but I've never quantified it into steps and given them actual, you know an easy to remember, you know, name like STOP and then the various you know components of that. So yeah, definitely be using that, passing it along to others. If you know, if they ask me, you know, how would I identify misinformation.”—ID246
	Preference for concise content	“If they're not—if they're short, sweet, and to the point, yes. ‐If they go on and on and on, no … I'm very, I'm pretty cut and dry kind of a person, you know.”—ID12
	Need for clarity and eliminating repetitiveness	“I almost think that you could probably get away with eliminating half of the spoken words, and still get the same message across. There are times so like when you would give an example like, oh you know, when you find something that's like, “oh, let's find a quick, easy dinner meal” or when you give examples that does help, it does help you understand. But like the first 2 slides in particular, it's just like a lot of info coming at you, and then after the first two slides, I feel like it gets a little bit more paired down.”—ID34
Video presentation and esthetic	Visual elements	“… It just looked dated to be honest with you as far as the templates, the slides, the powerpoint slide deck. It just looks dated, and you know, I would say, if there is something that is to be going out, I would have it's like a a watermark from the place where it's coming from, like [college], just to kind of give you credit‐credibility.”—ID235
	Suggestions for accessibility	“I would also make when you show the posts from social media. Maybe make the writing a little bit bigger for those that are sight impaired um. In you know, even mildly so.”—ID12
	Distracting animation	“The first video—the animated lady that they had on the side, she was very distracting on listening to her voice, because what she was telling me and what was on the screen was two different things. So now I'm trying to read that listen to her, and her movements were very distracting to me, because you're trying to hear this new information and kind of filtering it all in at the same time.”—ID9
	Desire for human narrator	“I don't like the cartoons. I guess, cause I'm a little older. And I just don't. They don't really like, pull me in. They're not very like empathetic, I guess. If that's the right word I'm looking for. But I would like to see a human on there discussing this stuff, I would lose track. I Would lose interest if it's just little animation. I Guess cartoon isn't the right word anymore but animation I'd rather see a person talking about it.”—ID8
Doctors as trusted sources	Reliability of doctors	“I might do some research, but I would be more inclined to believe what my doctor was uh forming a weight loss program for me, because it's detailed, not for me. I Believe that what's online is just like, almost like a cookie cutter. It could fit anybody. You have to know what fits for you. I Would be less inclined to believe what I see online. You know, against less than what my health provider.”—ID9

### 3.3 Self‐Efficacy and Confidence in Identifying and Handling Misinformation

After viewing the three videos, 57.1% of participants reported an increase in their self‐efficacy in identifying misinformation. Many participants rated their confidence as moderate to high (between 4–7 out of 7) both before and after viewing the videos, and they stated they are technologically savvy and competent when it comes to spotting misinformation. Despite this, participants felt that the STOP method materials reaffirmed their ability to spot misinformation and therefore reported increased scores. One participant stated: “*I feel like I'm pretty savvy on like identifying this kind of stuff but like*: *yeah*, *I feel like this gave … this does give like a helpful*. *helpful tool in general*”. Some participants explained their ability increased as they believe everyone can still be tricked by information online and there is always room to improve, as one participant stated: “*You know*, *the more information you have*, *the more you're able to make a conscious decision*, *you know*, *showing you whether it was good or bad that way you can make a better decision for yourself*.” Of the respondents who did not report an increased score, many initially rated their ability as a seven (out of 7). Therefore, there was no room for improvement among these individuals, though many stated that the videos better equipped them with a helpful strategy for identifying misinformation moving forward.

However, few participants reported increased self‐efficacy for how to handle misinformation after viewing the videos. Some participants had existing strategies that worked for them such as ignoring the inaccurate content, as one participant described: “*I just kinda like know to not engage with it. Really like I guess if*, *like*, *there's some specific action you want folks to take in regards to like reporting*, *misinformation or whatnot*, *then then maybe that isn't quite clear*, *but like to me*, *it just like*, *oh*, *like can't really* … *there's too much out there to just like get rid of it all*. *Just* … *people have to learn how to sift through it*.” Participants expressed that strategies for reporting or handling misinformation were not covered, and many stated their confusion regarding what the definition of “handling content” meant in the context of the interview; one participant argued: “*But I mean*, *what? What can you really do? Just ignore it*. *You can't really block it or report it*, *or anything*, *especially if it's on social media*. *I mean*, *you can respond to it*. *But you know*, *it's that's really pointless*.”

### 3.4 Feedback on the Stop Method

Overall, participants liked the STOP method and thought the presentation of the material served as a good introduction. Participants mostly agreed that the STOP method was easy to use and understand. They also felt that the way the material was presented was concise and helpful to grasp what the STOP method is and how to use it. Participants said they would recommend the STOP method to others and use it in the future when scrolling on social media. Many participants were able to think about its applicability to their lives and how other people they know may use it: “*I think that I will definitely utilize it and use it as like a good frame of reference whenever there is a post that I'm like*. “*Hmm! I wonder if that's true!*” *Being able to think through STOP*. *I think I'm I think I am like most likely to use it pretty often*, *like*, *if there's a post I'm questioning*. *I'll probably remember to think through that*.”

Throughout the interview, participants quickly grasped and implemented the STOP method. Additionally, participants felt they could easily and accurately apply it to the social media examples given in the three videos and talked through how they implemented the concept and how they would be on their own in the future. For example, one participant described how they would reference the STOP method when seeing a post in their own life “*It showed you*, *step by step*, *or what questions you should ask yourself when you see something*, *so if you ask yourself*, *if it falls in a category of three of those things that makes it wrong*, *then that's how you make a determination*.” However, some participants expressed their uncertainty that everyone would like the STOP method or find it personally useful: “*Mmm* … *maybe*. *I think it would depend on the circumstances*. *Yeah*, *you know*, *some people don't want all that*, *you know*. *People just want to hear*. *Go to your doctor or an RD. Get a good diet*. *Go to the gym*. *Talk to an exercise professional*, *you know*, *three sentences*. *‐Some people want all this others don't*. *So it it's be on a case by case basis*.” In addition, some participants expressed that adjustments could be made to the STOP method to make it stronger or more appealing to wider audiences. Improvements were suggested for how to present the STOP method by including additional specific examples of what to look out for in relation to each construct of the STOP method.

### 3.5 Video Content and Message Feedback

When asked about the videos' content and message, participants acknowledged that it was crucial to have evidence‐based health strategies presented in the videos and desired to learn accurate information about their health. Specially, participants desired videos focused on accurate information for personalized weight loss support: “*If they were more geared toward losing weight to better your health or losing weight for*, *women who've gone through menopause*, *or for senior citizens something that was more geared for my age*, *and where I am in my life as opposed to for everybody*.”

Furthermore, participants recognized that brief, concise content was an important feature. Specifically, the videos were more appealing if they provided a brief overview of the concept, which participants thought was enough to aid their online consumption while scrolling through the natural social media landscape. Additionally, participants advised researchers to revise the script in some places to be more concise and consistent across the three videos. Some participants felt there was extraneous information, with some offering specific alterations.

Participants were asked whether they thought the content and language of the videos were understandable and accessible. Overall, participants thought the information was easy to understand, informative, and effective: “*Yeah*, *I would say so*. *I don't know exactly your target audiences*, *but I think you know people who are trying to this way*, *and are usually like consumed by like social media*. *This could be a good way like the there was a lot of plain language used*, *I think, in my opinion*.” However, some individuals provided feedback for some script changes to improve the video content overall. Participants expressed that some messages were repetitive—especially regarding the social media examples and wanted more variety: “*I feel like the message was the same in each one*. *Just kind of be aware of false claims*, *and it just kept repeating itself*.”

### 3.6 Video Presentation and Aesthetics Feedback

Researchers inquired whether the video format was acceptable as opposed to other educational approaches (e.g., text, audio, etc.). Participants agreed that the video format was an ideal tool to provide supplemental health information. One participant said, “*I think videos are the best*. *Yeah*, *that way*. *You can watch them on your own schedule and take notes— better than better than a class*, *or anything written*.”

Researchers also asked for feedback on video appearance. Overall, the sample found the visual aspects of the video acceptable and pleasant. Feedback for visual aspects they wished were different, or suggestions for future alternations included the font and design, and the on‐screen animated narrator. For the font, participants liked the color‐coded text and slide backgrounds used across all three videos. Participants perceived these as satisfactory and beneficial to understanding the video content: “*No*, *I think it was good*. *It was very easily understood*. *Especially when you had the letters highlighted and the red kind of helps*. *Remember those and what they stand for*.” However, some interviewees advised researchers to change the font size for accessibility: “*I would also make when you show the posts from social media*. *Maybe make the writing a little bit bigger for those that are sight impaired um*. *In you know*, *even mildly so*.” Participants also suggested that there be improvements in scene transitions: “*Pieces*, *like one part like it ends at the talking*. *It's like immediately on the next line*. *It's like*, *could I use a little bit of a smoother transition*. *But I get it's kind of in progress*. *But I think overall*, *the content is good and whatnot*.”

For the on‐screen animated narrator, participants felt strongly that the animated character was a critical aspect of video design that needed to be revised. They provided feedback such as finding the animated narrator's movements distracting and a mismatch between the narration and the exact wording on screen. Overall, participants suggested that having several diverse characters or narrators (i.e., as opposed to the single character used) with varying races and ethnicities would be inclusive to broad audiences, including potentially using a real person/people instead of an animated narrator.

### 3.7 Doctors as Trusted Sources

Participants stated they would be more willing to watch the videos (i.e., videos about misinformation on social media) if they were recommended by their doctor. In general, participants believed that doctors are reliable sources of information. However, they would be more inclined to watch these videos to support their health if they had established trust and rapport with their doctor: “*Yeah*, *I think if it's like from the doctor*. *Specifically*, *then*, *I would totally like take time and watch them*, *and you know*, *try learn from it rather than seeing it on TikTok from an influencer*, *you know*.”

## 4 Conclusions

Overall, participants reported that the STOP Method was accessible and easy to use, and they expect that they will both use it to navigate weight loss information and would recommend it to others. They felt the videos effectively introduced the STOP method and its application and had minor suggestions to the script for improved clarity and video suggestions to add visual appeal and engagement. While many participants already had high self‐efficacy and pre‐existing strategies for handling inaccurate content online, there appears to be room for improvement in making the materials more robust with specific details about what to do once misinformation is identified.

The results of this exploratory interview study show promise for the use of a brief video‐based educational module to introduce the identification and handling of weight‐related misinformation online. Indeed, brief web‐based interventions have been found to be effective across a wide range of health behavior changes (e.g., immigrant susceptibility to misinformation, college student alcohol use) [26, 27]. While the STOP Method videos focused on education about how to identify misinformation, additional materials and the opportunity to practice may be needed to ensure that the materials have a lasting impact on information seeking and helping individuals to also handle the misinformation once spotted. A recent review of broad strategies to combat misinformation defined main categories of interventions, of which the STOP Method uses elements of inoculation and technique‐adoption at present; a future test and exploration of the method could explore its impact on real‐world social media use, an area that the review calls for as a critical place for future research [28]. This would entail measuring the impact of using the STOP Method on a participant's real social media use, including what information they share and comment on [28].

Participants also shared that they would be more likely to view videos such as those shared in this study if it was recommended by their doctor. Future research could explore how the videos could be available as a free‐standing online tool for patients who seek to use online resources to support their weight loss efforts and could be directly referred to use them by their doctor or other healthcare provider. As a next step, the STOP Method videos will be embedded in a primary care‐situated weight loss intervention (NCT06928571) as a tool to help patients navigate information they encounter in the context of online social communities focused on weight loss support.

While a relatively diverse sample of participants were interviewed (i.e., 35.7% male, 42.9% non‐White, age range 20–70 years), the majority of the sample had at least a Bachelor's degree and the reach may have been limited by the online recruitment sources we used (i.e., ResearchMatch, social media posts). Further, we were unable to draw conclusions about any subgroups within our sample due to their small numbers (e.g., by age group). More testing of the videos with an expanded sample will be an important next step to ensure that they are acceptable to a diverse audience. A recent review found that tailoring of misinformation interventions for culture and context may be key to their success, and that they are not necessarily universally effective [28]. Further, while we queried participants to understand if learning about the STOP Method in the span of the interview visit impacted their confidence in spotting and handling misinformation, they were unable to put the information to use in the real world. Additional pilot testing is needed to provide the videos and ask participants to apply it to their online health information seeking.

Given the high prevalence of overweight and obesity and the common use of online sources for health information, including social media, tools such as the STOP Method are of timely and critical importance. The public must be informed about how to spot and handle misinformation and be armed with tools to increase their confidence in finding high‐quality information on which to make health decisions for themselves and their families.

## Author Contributions

**Danielle E. Jake‐Schoffman:** conceptualization, methodology, writing – original draft, formal analysis, writing – review and editing. **Chrishann Walcott:** conceptualization, methodology, writing‐ original draft, formal analysis, writing‐review and editing. **Hannah A. Lavoie:** conceptualization, writing – original draft, formal analysis, writing – review and editing. **Francesca Wilkins:** writing – original draft, formal analysis, writing – review and editing. **Megan A. McVay:** conceptualization, writing – review and editing. **Montserrat Carrera Seoane:** conceptualization, writing – review and editing.

## Conflicts of Interest

The authors declare no conflicts of interest.

## Supporting information

Supporting Information S1

## References

[1] C. B. Martin , K. A. Herrick , N. Sarafrazi , and C. L. Ogden , Attempts to Lose Weight Among Adults in the United States, 2013–2016, 313, (2018), 1–8. 30044214

[2] G. L. Pappa , T. O. Cunha , P. V. Bicalho , et al., “Factors Associated With Weight Change in Online Weight Management Communities: A Case Study in the Loseit Reddit Community,” Journal of Medical Internet Research 19, no. 1 (January 2017): e17, 10.2196/jmir.5816. 28093378 PMC5282451

[3] X. Wang , R. A. Cohen . “Health Information Technology Use Among Adults: United States, July–December 2022 Key Findings Data From the National Health Interview Survey,” [Internet]. 2022, https://www.cdc.gov/nchs/products/index.htm.

[4] J. Chen and Y. Wang , “Social Media Use for Health Purposes: Systematic Review,” Journal of Medical Internet Research 23, no. 5 (May 2021): e17917, 10.2196/17917. 33978589 PMC8156131

[5] M. E. Waring , D. E. Jake‐Schoffman , M. M. Holovatska , C. Mejia , J. C. Williams , and S. L. Pagoda , “Social Media and Obesity in Adults: A Review of Recent Research and Future Directions,” Current Diabetes Reports 34, no. 18 (2018), 10.1007/s11892-018-1001-9. 29671135

[6] S. L. Pagoto , M. E. Waring , K. L. Schneider , et al., “Twitter‐Delivered Behavioral Weight‐Loss Interventions: A Pilot Series,” JMIR Research Protocols 4, no. 4 (October 2015): e123, 10.2196/resprot.4864. 26500186 PMC4704936

[7] R. An , M. Ji , and S. Zhang , “Effectiveness of Social Media‐based Interventions on Weight‐Related Behaviors and Body Weight Status: Review and Meta‐Analysis,” American Journal of Health Behavior 6, no. 41 (November 2017), 10.5993/AJHB.41.6.1. 29025495

[8] D. N. Cavallo , R. Martinez , M. Webb Hooper , and S. Flocke , “Feasibility of a Social Media‐Based Weight Loss Intervention Designed for Low‐SES Adults,” Translational Behavioral Medicine 11, no. 4 (April 2021): 981–992, 10.1093/tbm/ibaa070. 32716040 PMC8075609

[9] B. G. Southwell , E. A. Thorson , and L. Sheble , “The Persistence and Peril of Misinformation,” American Scientist 105, no. 6 (2017): 372–375, 10.1511/2017.105.6.372.

[10] D. Ramachandran , J. Kite , A. J. Vassallo , et al., “Food Trends and Popular Nutrition Advice Online—Implications for Public Health,” Online Journal of Public Health Informatics 10, no. 2 (September 2018), 10.5210/ojphi.v10i2.9306. PMC6194095 30349631

[11] V. Suarez‐Lledo and J. Alvarez‐Galvez , “Prevalence of Health Misinformation on Social Media: Systematic Review,” Journal of Medical Internet Research 23, no. 1 (January 2021): e17187, 10.2196/17187. 33470931 PMC7857950

[12] M. A. McVay , K. B. Cooper , M. L. Donahue , et al., “Engaging Primary Care Patients With Existing Online Tools for Weight Loss: A Pilot Trial,” Obesity Science & Practice 8, no. 5 (October 2022): 569–584, 10.1002/osp4.592. 36238223 PMC9535672

[13] D. E. Jake‐Schoffman , S. M. Carrera , K. Cooper , M. Rajoria , M. A. McVay , “Engaging Adults With Obesity in Organic Online Communities to Support Weight Loss: A Mixed Methods Pilot Study.” Psychol Health [Internet] 39, no. 4 (2024): 536–555, https://www.tandfonline.com/doi/abs/10.1080/08870446.2022.2087073. 10.1080/08870446.2022.2087073 35726546

[14] L. D. Scherer , J. McPhetres , G. Pennycook , et al. “Who Is Susceptible to Online Health Misinformation? A Test of Four Psychosocial Hypotheses.” Health Psychology [Internet] 40, no. 4 (March 2021): 274–284, https://pubmed.ncbi.nlm.nih.gov/33646806/. 33646806 10.1037/hea0000978

[15] W. Y. S. Chou , A. Oh , and W. M. P. Klein , “Addressing Health‐Related Misinformation on Social Media,” JAMA 320, no. 23 (December 2018): 2417–2418, 10.1001/jama.2018.16865. 30428002

[16] E. K. Vraga and L. Bode , “Using Expert Sources to Correct Health Misinformation in Social Media,” Science Communication 39, no. 5 (October 2017): 621–645, 10.1177/1075547017731776.

[17] L. Bode , E. K. S. VragaSomething , and S. Something , “Correction of Global Health Misinformation on Social Media,” Health Communication 33, no. 9 (September 2018): 1131–1140, 10.1080/10410236.2017.1331312. 28622038

[18] L. Bode and E. K. Vraga , “Related News, That Was Wrong: The Correction of Misinformation Through Related Stories Functionality in Social Media,” Journal of Communication 4, no. 65 (June 2015): 619–638, 10.1111/jcom.12166.

[19] Kozyreva A. , Lorenz‐Spreen P. , Herzog S. M , et al. “Toolbox of Individual‐Level Interventions Against Online Misinformation.” Nature Human Behavior [Internet] 8, no. 6 (June 2024): 1044–1052, https://www.nature.com/articles/s41562‐024‐01881‐0. 10.1038/s41562-024-01881-0 38740990

[20] Blakeslee S. , “The CRAAP Test.” [Internet]. LOEX Quarterly, (2004), https://commons.emich.edu/loexquarterly/vol31/iss3/4.

[21] CRAAP Test | EBSCO Research Starters [Internet], https://www.ebsco.com/research‐starters/social‐sciences‐and‐humanities/craap‐test.

[22] C. B. Weir and A. Jan , BMI Classification Percentile and Cut Off Points. StatPearls. 2022. Treasure Island, FL (StatPearls Publishing, 2023). 31082114

[23] G. Guest , A. Bunce , and L. Johnson , “How Many Interviews Are Enough?: An Experiment With Data Saturation and Variability,” Field Methods 18, no. 1 (2006): 59–82, 10.1177/1525822x05279903.

[24] B. Glaser and A. L. Strauss , The Discovery of Grounded Theory: Strategies for Qualitative Research (Aldine Publishing Company, 1967).

[25] J. Corbin and A. Strauss , Basics of Qualitative Research (Sage, 2015).

[26] M. Carrera Seoane , D. E. Jake‐Schoffman , A. R. Mobley , and M. A. McVay , “Addressing Susceptibility to Non‐Prescription Substances for Weight Loss Among Immigrant Hispanic College Students: A Pilot Study,” Journal of Immigrant and Minority Health 27, no. 1 (2024): 62–73, 10.1007/s10903-024-01632-3. 39312059

[27] R. F. Leeman , E. Perez , C. Nogueira , and K. S. DeMartini , “Very‐Brief, Web‐Based Interventions for Reducing Alcohol Use and Related Problems Among College Students: A Review,” Frontiers in Psychiatry 6 (2015): Frontiers Media S.A., 10.3389/fpsyt.2015.00129. PMC4585336 26441690

[28] Kozyreva A. , Lorenz‐Spreen P. , Herzog S. M , et al. “Toolbox of Individual‐Level Interventions Against Online Misinformation.” Nature Human Behavior [Internet] 8, no. 6 (June 2024): 1044–1052, https://pubmed.ncbi.nlm.nih.gov/38740990/. 10.1038/s41562-024-01881-0 38740990

