# Supplementary material for: Exploring the Acceptability of the STOP Method for Addressing Weight Loss Misinformation on Social Media: An Interview Study
Source: Obes Sci Pract. 2025 Jun 21;11(3):e70080. doi: 10.1002/osp4.70080 (PMC12182187; doi:10.1002/osp4.70080)
Supplement: Supplementary file 1 — Supporting Information S1 [file OSP4-11-e70080-s001.docx]

**Supplemental Material**

**Semi-Structured Interview Script**

**NOTE:** Conduct informed consent first – participants must give permission before beginning

Introduction:

** Start recorder ** [Do not transcribe introduction]

Thank you for coming, again my name is Chrishann and I will be asking you a few questions that are relevant to our research study. Our goal today is to get your perspective on a few videos we are developing. We welcome any of your thoughts and hope to use your opinions to improve the materials that we will be showing you today. We will be asking you some broad questions and some very specific questions about the scripts and the visuals that you will see on-screen.

One thing we want to emphasize is that these videos are a rough draft. We plan for the final versions to be more polished. But any feedback you give us at this stage allows us to improve it.

**Prior Experience with Health Misinformation on Social Media**

- Before we dive into the videos, I want to ask you a couple of quick questions about health information online.

1. Please rate your confidence on a scale from 1 to 7, with 1 being "not at all confident" and 7 being "very confident" for the following two statements.
   - I am confident that I can recognize or identify if health information I see online is false or inaccurate.
   - I am confident that I know what to do if I see health information online that is false or inaccurate.
2. **Ok, next I will be asking you to imagine the following scenario**: Prior to an appointment with your doctor, you received an email asking if you want to lose weight and you say “yes”.. You attempt to find online support and accurate information through social media to help you reach your weight loss goal.

Then, you are told your doctor would like you to watch a few short videos and ask some questions followed by personalized feedback. What thoughts would you have about this?

- - Do you think these videos would be useful as you are interacting with online support groups?
  - How likely are you to follow your doctor’s recommendations to watch the videos? Why?

1. We are now going to have you go through some brief videos. You can take notes as thoughts come to mind. We will then discuss final thoughts AFTER all videos. Once you are finished, we’ll ask you for any feedback you have. You can tell us about things you liked and didn’t like, things that you had difficulty understanding, or any other thoughts that come to mind. After that, we will go back through each part of it again and ask you some specific questions.
2. Ok, let’s get started. What are some of your thoughts about the videos?
   - What is something you liked about this?
   - What is something you didn’t like?  o What is something you didn’t understand?
   - What would you change about this if you could?)
   - How did you find the length of the script? (Was it too short, too long, or just enough information?)
3. Now we are going to go through each video individually and ask specific questions. For each video:
   - How would you describe the purpose of this video? (Rephrased in case they need clarification:
   - Ware your thoughts on this video?
   - Were there parts you liked? Parts you didn’t like?
   - What might you change about this video?
   - How did you find the length of this video? Did you want more or less information on this topic?
   - Any other thoughts on the video before we move on?
4. Now, I have a couple of final questions. Please rate your confidence on a scale from 1 to 7, with 1 being "not at all confident" and 7 being "very confident" for the following two statements.
   - **Statement 1**: I am confident that I can recognize or identify if health information I see online is false or inaccurate.
     - (If needed) Again, please rate your confidence from 1 to 7, with 1 being "not at all confident" and 7 being "very confident." You can choose any number from 1 to 7.
   - **Statement 2**: I am confident that I know what to do if I see health information online that is false or inaccurate.
5. (If needed) Again, please rate your confidence from 1 to 7, with 1 being "not at all confident" and 7 being "very confident."
   - - - Okay great! Thank you so much for your answers.
       - Okay great! Thank you so much for your answers.

- Okay great! Thank you so much for your answers

1. After watching these videos, do you feel any different about your ability to spot or identify and handle or deal with misinformation on social media? Why or why not?

**Wrap-Up**

- Is there anything else about the videos or anything else we talked about today that you’d like to share with me?

*****End transcription*****

Thank you again for all of your input. Now I am going to ask you a few descriptive questions; I’ll write down your answers as we go. Lastly, I’ll collect your information so that we can administer your gift card.
